# How does social support affect subjective well-being among Chinese widowed older adults?

**DOI:** 10.3389/fpubh.2024.1352585

**Published:** 2024-03-04

**Authors:** Jun Zhang, Fangyuan Cao, Chao Yang

**Affiliations:** ^1^School of Marxism, Chang'an University, Xi'an, China; ^2^School of Social and Public Administration, East China University of Science and Technology, Shanghai, China

**Keywords:** subjective well-being, widowed older adults, social support, guardian, rural, urban

## Abstract

**Background:**

Social support (SS) is an important factor influencing subjective well-being (SWB) in older adults. This is especially true for the special group of widowed older adults (WOA). Widowhood means that older adults have lost their most important SS, and therefore, the search for a guardian from outside the spouse becomes a central issue in ensuring the SWB of WOA.

**Methods:**

The data for this paper were obtained from CGSS 2021, a large national social survey in China. We operationalized SWB as an individual’s overall perception of his or her experience of happiness using ‘affective well-being’ (i.e., emphasizing an individual’s positive affective experiences), and scores were calculated using a Likert scale. This study used linear regression modeling to examine the impact of SS on the SWB of WOA (aged 60 and above).

**Results:**

It was found that, first, this study presents the role of different circles of SS on the SWB of WOA, fully highlighting the importance of social context. Specifically, daughters, neighbors, and relatives constitute the guardians of the SWB for WOA in rural, whereas daughters and friends constitute the guardians of the SWB for WOA in rural. Second, the protective resources provided by the guardians not only serve as a buffer for WOA in distress but also reduce the likelihood of negative events occurring, thereby increasing WOA’s SWB.

**Discussion:**

This paper partially corroborates the findings of established studies on the topic of SS and SWB among older adults and the above findings not only help us to further explain the relationship between SS and SWB theoretically but also help us to rationalize the construction of SS for WOA practically.

## 1 Introduction

In the era of population aging, SWB has become an important condition for evaluating social life quality alongside the level of economic development and social development (1–3). As a protective force associated with all-cause mortality, the increase of SWB is significantly associated with the reduction of mortality in the general population (especially for the older adult) (4, 5), and it has a protective effect on the reduction of mortality and disease incidence in the older adult (6). Not only that, elevated SWB in older adults also helps to reduce the risk of developing some related diseases (e.g., stroke, diabetes, cardiovascular-metabolic diseases, and upper respiratory diseases). Therefore, the study of SWB in older adults has become a hot topic in sociology, psychology, public policy, and other fields in the last 20 years (3, 6).

Among the many predictor variables of SWB, marital status is generally recognized as the key factor with the most significant impact (2), with marriage positively contributing to SWB in both spouses, and numerous empirical studies confirming that the association is strong, cross-cultural, and cross-geographical (7–9). Empirical studies have found that compared to married and divorced individuals, widowed individuals report the lowest SWB scores, and the highest loneliness and negative mood scores and that WOA report significantly lower SWB scores than older adult with spouses (10–12). Therefore, for WOA, finding protective resources from sources other than their spouses becomes crucial to safeguard their physical and mental well-being. Unfortunately, despite a range of results in existing research on SWB in older adults, studies have mainly focused on exploring the negative utility of SWB in older adults, and studies on WOA as a specific group are scarce (1). Moreover, previous studies on SS on SWB usually regard SS as a holistic variable, ignoring the typology and specificity of SS, and failing to discover the heterogeneity of the role of SS in influencing the pathway of SWB.

In conclusion, it is urgent to explore the subjects that may become protective resources for WOA’s SWB from the perspective of SS and to construct an SS system for the older adult promptly to enhance WOA’s well-being. Therefore, this study attempted to categorize SS in older adults into three types based on the Convey Model and to investigate the effects of SS on SWB of WOA in China using data from the China General Social Survey (CGSS2021). Thus, it further advances the understanding of the relationship between SS and SWB and contributes in some way to safeguarding and improving the SWB of WOA at the practical level.

## 2 Literature review

### 2.1 Convoy model and SWB

Resource conservation theory suggests that a change in marital status is one of the most significant changes in an individual’s life course, and the loss of a spouse is one of the most difficult and traumatic events, with older adults needing more time and energy to adjust and adapt to this change than in the case of divorce. In addition, widowhood also means that older people are deprived of stable long-term emotional and functional support resources, and the loss of these protective resources means that individuals face more difficulties in coping with negative events, which in turn reduces their level of mental health (13, 14). In other words, individuals are guarded and accompanied by significant others throughout their lives, including not only those with whom they have intimate relationships but also those who have a key influence on their lives (15). Throughout an individual’s life course, interactions with significant others are always characterized by longevity and stability, and inter-individual relationships evolve (16); for example, parent–child relationships remain close from childhood to old age, and husband-wife, relative, and friend relationships are relatively stable throughout the entire life course, and influence an individual’s SWB through the accumulation of interactions (15). The accumulation of interactions with significant others allows these relationships to form part of an individual’s SS, which can provide SS, including emotions, information, friendship, care and evaluation, and so on, and thus act as guardians of SWB during their adulthood and old age (17).

The convey model emphasizes the different types and levels of characteristics of escorts, which are based on the closeness of the escort to the individual and independence of the social role, resulting in three circles, core circle, middle circle, and outer circle, with the individual residing at the center of these circles. Specifically, the core circle consists of relationships that provide the most SS, usually including family members such as spouses and children, who are the individual’s subjectively closest relationships and do not change according to the individual’s social role, and who are often the most important guardians of the individual’s SWB. The middle circle usually consists of relationships such as relatives and friends, who also provide important SS to the individual, however, the middle circle will change to some extent based on the individual’s changing social role in adulthood. The outer circles are the least close to the individual compared to the previous two circles and can be entirely dependent on changes in the individual’s social role, but they still provide the individual with essential SS resources at certain times in the life course (17, 18). The three circles mentioned above have different structural characteristics, such as differences in size, frequency of interactions, and types of SS, which makes them have different effects on SWB in older adults in different social contexts. Empirical studies have found that among the multiple dimensions of SS, emotional support is the most predictive of an individual’s SWB (19), and the above findings have also been confirmed in the older adult population (20). Synthesizing the convoy model with established empirical findings, this study argues that after losing protective resources from spouses, significant others such as children, relatives, and friends constitute guardians for WOA’s SWB.

### 2.2 Core circle and SWB of WOA

Antonucci’s study found that older adults over the age of 60 were more likely to nominate their spouses and adult children than their parents as guardians of their core circle (18, 21), so adult children became a major component of the core circle after the loss of a spouse. Therefore, child characteristics have become an important predictor of SS resources and mental health in older parents. Logan’s study showed that the main process of intergenerational exchange is the financial support provided by adult children to older adult parents, which is one-third of the total income of older adult parents, so the number of children has an important impact on securing the old age of the older adult (22). Studies based on intergenerational support for rural older adults have found that an increase in the number of children brings more SS to rural older adults (23). The contribution of children to the SS of older people makes them important guardians of older people’s SWB. Related studies have found that the number of children has a significant positive correlation with the SWB of older adult parents (24), and in addition, companionship support from children can significantly enhance the SWB of WOA (25).

Related studies have shown that the number of children and SWB of the older adult are closely related. In South Korea, the self-rated well-being of older adult people with many children is higher than that of older adult people with a single child (26). In China, having children as well as multiple children significantly increased the SWB of the older adult (27), and the effect of the number of children on the SWB of the older adult group was more obvious, and the increase in the number of children could significantly increase the SWB of the rural older adult. In particular, when the parents are over 60 years old, the happiness index of multiple-child families will overtake that of one-child families (28, 29). However, there is some controversy about the effect of the number of children on parents’ physical and mental health, and related studies have shown that children themselves are a source of parental stress, increased financial costs, and physical pain and that the effect of child quality on parental SWB is more robust than that of the number of children (30–34). From the perspective of SS, for older adults, financial support, emotional comfort, and life care from their children are key factors influencing their SWB. Studies in Europe and the United States have found that when supporting parents, sons are more likely to provide financial support and daughters are more likely to provide life care (35). Similarly, in contemporary Chinese families, research on intergenerational support suggests that there may be child-sex differences in the type of SS provided by adult children (36, 37). Especially in rural areas, where most parents do not have stable financial resources, the financial support of sons becomes increasingly important. However, both the financial support provided by sons and the life care provided by daughters are beneficial in increasing the SWB of older people. In short, an increase in the number of children increases the likelihood that older adults will receive more SS, which in turn increases their SWB. Therefore, considering the role of adult children as guardians of the WOA and the SS provided by sons and daughters to their older adult parents, based on the above analysis, the following research hypotheses are proposed:

*Hypothesis* 1: An increase in the number of daughters will significantly increase the SWB score of WOA.

*Hypothesis* 2: An increase in the number of sons will significantly increase the SWB score of WOA.

### 2.3 Middle circle and SWB of WOA

Based on the criteria of closeness of the escort to the individual and independence of social roles, relatives and friends are in the middle circle. In the case of relatives, the closeness of relatives to the WOA is less affected by the change in the individual’s social role due to the existence of blood ties. Usually, relatives and WOA have a high degree of consistency in family networks and upbringing, which makes them share similar family concepts, behavioral patterns, and values; moreover, relatives can provide higher interpersonal trust and reciprocity during the interaction process (38), and thus interactions with relatives can help to elicit positive affective experiences, which can, in turn, increase the SWB scores of WOA. Research on survey data of older adults in Taiwan suggests that interactions with relatives can help improve the SWB scores of older adults (39).

Friendships have different meanings for older adults compared to family ties: firstly, friendships are acquired relationships, an achievement that individuals work hard to maintain; secondly, there is a higher degree of structural similarity between friends and individuals in the same age group; and lastly, friendships can expand older adults’ social networks, and thus their SS (40). Friends tend to interact based on pleasure, and during social interactions, friends can provide more respect, tolerance, acceptance, and mutual understanding, as well as provide immediate and timely feedback regarding the exchange of pleasure and meaning (41), Interaction with friends through leisure and recreation is considered an important SS resource (42). SS from friends often serves as a buffer to effectively cope with and alleviate negative emotions when older adults encounter negative life events, and empirical studies have found that interactions with friends help to enhance SWB in older adults.

Widowhood leads to the loss of important protective resources such as optimism, interpersonal dependence, reciprocity, understanding, and acceptance (43), thus increasing their difficulties in coping with negative events and emotions. Relatives and friends constitute an intermediate circle of caretakers for WOA, and the SS gained by interacting with them can help to cope with the above situation and enhance the SWB of WOA. Therefore, we propose the following research hypothesis:

*Hypothesis* 3: Increased frequency of interaction with relatives significantly increases SWB scores in WOA.

*Hypothesis* 4: Increased frequency of interaction with friends significantly increases SWB scores of WOA.

### 2.4 Outer circle and SWB of WOA

There is a proverb in China, Better is a neighbor. The phrase means that in the event of an emergency where help is needed, distant relatives are often not able to help in the same timely manner as close neighbors. So, neighbors are an important source of SS for WOA and are in the outer circle. For example, when we need help, our neighbors will not hesitate to lend a helping hand; when we hold family gatherings, our neighbors will participate enthusiastically and make the gatherings livelier; when we encounter problems in the community, our neighbors will discuss solutions together and work together to maintain harmony in the community. These conveniences and cozy atmosphere will undoubtedly improve the quality of life of the older adult, thus enhancing their SWB. Compared with other age groups, the role of the neighbor as a guardian of older people’s SWB is more obvious: firstly, lower social mobility makes neighbor relationships more long-term and stable for older people; secondly, due to the geographical proximity, neighbors can not only provide immediate information about local life during interaction but also provide the first help when older people encounter emergency problems (44); and finally, due to the warmth experienced by the neighbor, most older people can maintain good relationships and obtain stable SS from neighbor interactions. It has been found that SS from neighbors can increase self-esteem and reduce depression and loneliness and that neighbor relationships have become an important way for WOA to obtain emotional support. Based on the importance of neighbor support, previous studies have suggested that interactions with neighbors can help to increase older people’s SWB (45, 46).

Over the past 30 years, a large number of empirical studies have supported the positive impact of religious participation on SWB in people from different countries (47, 48). Participation in religious activities helps to enhance older people’s perceptions of the meaning of life and provides them with a sense of purpose through collective psychological identity, which in turn provides them with a sense of security and stability (49, 50). On one hand, it can help WOA adapt to life after losing their spouses, and on the other hand, it can also help them rebuild their sense of meaning and value of life, as well as obtain SS from their peers, which are of great significance in safeguarding the SWB of WOA (51). For example, many religious practices emphasize loving kindness and encourage people to pay attention to the needs and feelings of others. Such attention and love can enhance the bond between people and thus promote SWB. As another example, many religious activities will emphasize inner peace. Through methods such as meditation and prayer, people can relax their bodies and minds and enhance self-control and emotional regulation, thereby reducing stress and enhancing SWB.

Compared with the core circles and middle circles, the SS provided by the neighbor and religious activities has a special function as a guardian for the WOA, and the neighbor interaction and participation in religious activities can help the WOA alleviate their negative emotions and adapt to the current state of life in a relatively short period, which leads to the following research hypothesis:

*Hypothesis* 5: An increase in the frequency of interaction with neighbors significantly raises SWB scores among WOA.

*Hypothesis* 6: An increase in the frequency of participation in religious activities significantly increases SWB scores among WOA.

Since the reform and opening up, although China has made great achievements in economic development, there is still a huge urban–rural gap in development. This gap is mainly reflected in many aspects of SS, such as income, education, health care, consumption, employment, and government public investment (52). Therefore, the place of residence, as an important socio-economic factor, is an important variable affecting the SWB of older adults (53, 54). Relevant studies have shown that the level of well-being of urban older adult is significantly higher than that of rural older adult (55). Specifically, the urban area is a more modern social field, where health protection, medical resources public transportation, etc. are usually superior, and the older adult can more easily obtain higher income and quality medical services, etc., which may play a positive role in promoting their SWB. However, due to the faster pace of urban life and more detached social relationships, the older adult may face problems such as estrangement of family and neighbors, which may have some negative impact on their SWB (54). The rural area is a more traditional social field, where kinship and neighborly relations are closer, and the older adult may be more likely to receive family care and more companionship and interaction, which may have a positive impact on their SWB. However, due to the limitations of the natural environment and economic fundamentals, the rural older adult face problems such as meager income, lack of medical resources, and inconvenient transportation, which may have a certain negative impact on their SWB (53). To sum up, considering that the dual characteristics of urban and rural areas in China are still obvious, we propose the following hypothesis:

*Hypothesis* 7: The effect of SS on SWB is different between urban and rural areas.

## 3 Materials and methods

### 3.1 Data

The data used in this paper was from the Chinese General Social Survey (CGSS2021), which was collected and published by the China Survey and Data Center of Renmin University of China, and uses a multi-stage stratified sampling method and structured interviews to collect data covering all provincial administrative units in mainland China. The CGSS data is highly consistent with international data such as the World Value Survey in its measurement of SWB, and covers key variables such as SS, socioeconomic status (SES), health status, and demographic factors, making it highly relevant to the research topic of this paper. With the respondent’s consent, the enumerator provides a questionnaire to measure SWB. The enumerator provides a questionnaire that consists of detailed questions about the respondent’s SES. Through screening, the CGSS2021 data’s effective sample size was 11,354, and by research convention, we defined the age range of the sample as 60 years and above (China’s Law on the Protection of the Rights and Interests of the Older adult stipulates that the starting standard for the age of the older adult is 60 years), thereby deleting 7,467 samples aged less than 60 years. Of the remaining 3,887 samples, we went on to delete 2,938 samples of older adult with spouses with marital status and 258 samples that did not respond to the information on SS, resulting in 691 samples that entered the analytical model for this paper.

### 3.2 Variables

#### 3.2.1 Dependent variables

The dependent variable is SWB. In this study, we used Diner’s classic definition of SWB, which is “the overall perception of how people measure their lives in the present or a past period” (56). Concerning previous research on SWB in older adults (1, 57), we also operationalized SWB as an individual’s overall perception of his or her experience of happiness using ‘affective well-being’ (i.e., emphasizing an individual’s positive affective experiences), and scores were calculated using a Likert scale. The questionnaire measured SWB through the question “Overall, do you feel that you are happy in your life?,” which was coded as a continuous variable (1 = very unhappy, 5 = very happy), with higher scores representing greater happiness.

#### 3.2.2 Independent variables

##### 3.2.2.1 Number of children

Based on the operationalization of the question “How many children do you have?” in the questionnaire, the “Number of sons” and “Number of daughters” reported by the respondents were coded as continuous variables.

##### 3.2.2.2 Relatives’ interactions

The questionnaire asked respondents to report “In the past year, how often did you meet with relatives who did not live with you in your free time?” (1 = never, 5 = daily), We coded respondents’ answers based on a Likert scale, the higher scores, the higher interactions.

##### 3.2.2.3 Friends’ interaction

Based on the question “How often do you engage in social and recreational activities with other friends?” (1 = never, 7 = almost every day), we coded the responses answers based on a Likert scale, the higher scores, the higher interactions.

##### 3.2.2.4 Neighbor interaction

Based on the question “How often do you engage in social and recreational activities with your neighbors?” in the questionnaire (1 = never, 7 = almost every day), we operationalized the variables in a coding manner consistent with “friends’ interactions.”

##### 3.2.2.5 Religious activities

Based on the question “How often do you participate in religious activities?” we coded the responses answers based on a Likert scale (1 = never, 9 = a few times a week), the higher scores, the higher interactions.

#### 3.2.3 Control variables

In addition to marital status, previous studies have examined the factors influencing SWB in older adults mainly in terms of SES, health status, demographic factors, and social relationships (1, 9, 13, 58). Therefore, we followed the research convention of including the above factors as control variables in the model. These variables include gender (binary-variables, 0 = Male, 1 = Female), age (continuous variables, the range is 60–94), schooling years (continuous variables, the range is 0–11), Annual income (continuous variable, natural logarithm, the range is 0–10.82), Party membership (binary-variables, 0 = Others, 1 = CPC member), self-assessed physical health (multi categorical variables, 1 = very unhealthy, 5 = very healthy) and relative SES (multi categorical variables, 1 = lower, 3 = higher).

### 3.3 Analysis strategy

Similar to previous research on SWB in older adults (58), this study used SPSS Statistics 24.0 and linear regression modeling to test the above research hypotheses. In addition, considering the differences in social structure, cultural traditions, and interaction patterns between urban and rural, This study chose to divide the total sample into rural and urban samples and tested the research hypotheses through stepwise regression modeling: The first step is to build a baseline model with control variables; the second step is to build an analytical model with core independent variables; and the third step is to build a composite model with both control variables and core independent variables to examine the role of SS in the three circles on the SWB of WOA after controlling for SES, self-assessed health status, and relevant demographic factors.

We used the OLS model to validate the effect of SS on SWB in WOA, as shown in Equation 1:


(1) $$y=b_{0}+b_{1}x_{1}+b_{2}x_{2}+\ldots +b_{n}x_{n}$$

Where Y is the explanatory variable, i.e., SWB; X_n_ is the independent variable that affects the change of Y; b_0_, b_1_, b_2_...bn are the corresponding regression summations, which are generally calculated by statistical analyses.

## 4 Results

### 4.1 Descriptive statistical analysis

The results of the descriptive statistics are shown in Tables 1, 2. Based on the way the sample was divided in this study, WOA accounted for 22% of the total number of older adults (*n* = 3,141), a proportion that is relatively similar to the predicted proportion (21%) based on national census data. The mean SWB scores for rural and urban WOA were 3.75 and 3.99, respectively, which were lower than those of rural older adult with spouses (3.89) and urban (4.04), which is in line with previous studies on the relatively lower levels of SWB among Chinese WOA.

**Table 1:** Statistical table describing basic variables for rural WOA (*N* = 389).

Variable	Range	Frequency	Mean/percentage	SD	Details
SWB	1–5	389	3.75	0.92	Multi-categorical variables,1 = very unhappy, 2 = relatively unhappy, 3 = average, 4 = relatively happy, 5 = very happy
SS					
Number of daughters	0–7	389	1.36	1.24	Continuous variables
Number of sons	0–8	389	1.77	1.16	Continuous variables
Relatives’ interaction	1–5	389	2.02	0.79	Continuous variables 1 = never, 2 = several times a year or less, 3 = several times a month, 4 = several times a week, 5 = daily
Friends’ interaction	1–7	389	4.05	2.20	Continuous variables 1 = never, 2 = one time a year or less, 3 = a few times a year, 4 = about once a month, 5 = a few times a month,6 = one to two times a week,7 = almost every day
Neighbor interaction	1–7	389	5.36	1.80	Continuous variables 1 = never, 2 = one time a year or less, 3 = a few times a year, 4 = about once a month, 5 = a few times a month,6 = one to two times a week,7 = almost every day
Religious activities	1–9	389	1.49	1.38	Continuous variables 1 = never, 2 = less than 1 time a year, 3 = about 1 to 2 times a year, 4 = a few times a year, 5 = about 1 time a month, 6 = 2 to 3 times a month, 7 = almost every week, 8 = every week, 9 = a few times a week
Gender					Binary-variables
Female		261	67%		1 = Female
Male		128	33%		0 = Male
Age	60–94	389	72.64	7.68	Continuous variables
Party membership					Binary-variables
Party		16	4%		1 = CPC member
Non-Party		373	96%		0 = Others
Annual income	0–10.82	389	5.85	3.74	Continuous variables
Relative SES (Reference: peers)	1–3	389	1.62	0.57	Multi-categorical variables,1 = lower,2 = the same,3 = higher
Relative SES (Reference: 3 years ago)	1–3	389	2.23	0.63	Multi-categorical variables,1 = lower,2 = the same,3 = higher
Schooling years	0–11	389	2.53	3.04	Continuous variables
Health status	1–5	389	3.02	1.10	Multi-categorical variables,1 = very unhealthy, 2 = relatively unhealthy, 3 = average, 4 = relatively healthy, 5 = very healthy

**Table 2:** Statistical table describing basic variables for urban WOA (*N* = 302).

Variable	Range	Frequency	Mean/percentage	SD	Details
SWB	1–5	302	3.99	0.78	Multi-categorical variables,1 = very unhappy, 2 = relatively unhappy, 3 = average, 4 = relatively happy, 5 = very happy
SS					
Number of daughters	0–6	302	1.21	1.08	Continuous variables
Number of sons	0–6	302	1.45	1.10	Continuous variables
Relatives’ interaction	1–5	302	2.30	0.89	Continuous variables 1 = never, 2 = several times a year or less, 3 = several times a month, 4 = several times a week, 5 = daily
Friends’ interaction	1–7	302	3.95	2.08	Continuous variables 1 = never, 2 = one time a year or less, 3 = a few times a year, 4 = about once a month, 5 = a few times a month,6 = one to two times a week,7 = almost every day
Neighbor interaction	1–7	302	4.60	2.10	Continuous variables 1 = never, 2 = one time a year or less, 3 = a few times a year, 4 = about once a month, 5 = a few times a month,6 = one to two times a week,7 = almost every day
Religious activities	1–9	302	1.76	1.86	Continuous variables 1 = never, 2 = less than 1 time a year, 3 = about 1 to 2 times a year, 4 = a few times a year, 5 = about 1 time a month, 6 = 2 to 3 times a month, 7 = almost every week, 8 = every week, 9 = a few times a week
Gender					Binary-variables
Female		235	78%		1 = Female
Male		67	22%		0 = Male
Age	60–94	302	74.13	8.26	Continuous variables
Party membership					Binary-variables,
Party		61	20%		1 = CPC member
Non-Party		241	80%		0 = Others
Annual income	0–12.90	302	9.41	2.47	Continuous variables
Relative SES (Reference: peers)	1–3	302	1.74	0.58	Multi-categorical variables,1 = lower,2 = the same,3 = higher
Relative SES (Reference: 3 years ago)	1–3	302	2.12	0.59	Multi-categorical variables,1 = lower,2 = the same,3 = higher
Schooling years	0–18	302	6.08	4.44	Continuous variables
Health status	1–5	302	3.21	0.96	Multi-categorical variables,1 = very unhealthy, 2 = relatively unhealthy, 3 = average, 4 = relatively healthy, 5 = very healthy

Regarding SS, in the core circle, the number of sons (1.77) and number of daughters (1.36) are higher among rural WOA compared to urban WOA (1.45 and 1.21, respectively), and further descriptive statistics show that 52% of rural WOA compared to 41% of urban WOA have two or more sons, and 40% of rural WOA compared to 31% of urban WOA have two or more daughters, and most rural and urban WOA live in extended families (85 and 74%, respectively). 31% of urban WOA had two or more daughters, and the majority of rural and urban WOA lived in large families (85 and 74% respectively). In the middle circle, further analysis shows that most WOA interact with relatives “a few times a year or less”; more than half of WOA interact with friends “about once a month” or more. In the outer circle, the results show that the frequency of interaction with neighbors is higher among rural WOS (5.36) than urban WOS (4.60), and further analysis shows that 35.73% of rural WOS interact with their neighbors “almost every day”; The frequency of participation in religious activities was lower for both rural and urban WOS (1.49 vs. 1.76), and the percentage of older persons who participated in religious activities was 15% (rural) vs. 19% (urban), respectively.

### 4.2 Regression results and analysis

The results are shown in Table 3. By the analytical strategy, we first establish the benchmark model 1 and model 3 which cover all the control variables, then we establish model 2 and model 4 which only include the core independent variables, and finally, we establish the comprehensive model 3 and model 6 which include the control variables and the core independent variables. The above models passed the significance test as a whole, and none of them has a covariance problem.

**Table 3:** OLS model of the effect of SS on SWB.

Variable	Rural			Urban		
	Model 1	Model 2	Model 3	Model 4	Model 5	Model 6
	b/S.E.	b/S.E.	b/S.E.	b/S.E.	b/S.E.	b/S.E.
Gender (Reference: male)	0.249*		0.193	0.183		0.147
	(0.101)		(0.101)	(0.105)		(0.106)
Age	0.003		−0.002	0.012*		0.010
	(0.006)		(0.006)	(0.005)		(0.006)
Party membership (Reference: others)	0.054		0.118	0.255*		0.227
	(0.254)		(0.251)	(0.118)		(0.117)
Annual income	−0.002		−0.009	0.003		0.007
(logarithmic)	(0.012)		(0.012)	(0.017)		(0.017)
Relative SES (Reference: peers)	0.391***		0.350***	0.266***		0.244**
	(0.081)		(0.081)	(0.077)		(0.077)
Relative SES (Reference: 3 years ago)	0.157*		0.150*	0.269***		0.271***
	(0.072)		(0.070)	(0.070)		(0.071)
Schooling years	0.024		0.022	−0.010		−0.009
	(0.016)		(0.016)	(0.011)		(0.011)
Health status	0.219***		0.208***	0.155***		0.136**
	(0.042)		(0.041)	(0.044)		(0.044)
Number of daughters		0.078*	0.078*		0.114**	0.083*
		(0.037)	(0.036)		(0.041)	(0.042)
Number of sons		0.014	0.014		0.096*	0.034
		(0.040)	(0.039)		(0.041)	(0.042)
Relatives’ interactions		0.211***	0.170**		0.132**	0.089
		(0.059)	(0.055)		(0.051)	(0.049)
Friends’ interaction		−0.006	−0.020		0.050*	0.053*
		(0.024)	(0.022)		(0.025)	(0.024)
Neighbor interaction		0.091**	0.064*		0.008	−0.015
		(0.029)	(0.027)		(0.025)	(0.023)
Religious activities		0.021	0.015		0.007	0.007
		(0.034)	(0.031)		(0.025)	(0.023)
Constants	1.706**	2.704***	1.478**	1.420**	3.160***	1.158*
	(0.524)	(0.206)	(0.537)	(0.505)	(0.173)	(0.527)
Adjusted R-squared	0.198	0.066	0.231	0.175	0.067	0.197

*significant at *p* < 0.05. **significant at *p* < 0.01. ***significant at *p* < 0.001.

Regarding the control variables, the effect of annual income on the SWB of WOA fails the significance test, while the improvement of relative SES has a significant positive driving effect on the SWB of both urban and rural WOA, and the above results are consistent with the findings of previous studies on relative SES and residents’ SWB (59). Improvement in self-assessed health significantly increases the mean score of SWB for both urban and rural WOA, and the data results support the findings of previous studies on the high correlation between self-assessed health and SWB for older adults (11). For WOA, the effect of schooling years on the dependent variable did not pass the statistical test of *p* < 0.05, a result that is also consistent with Kaufman’s study (60). The role of gender on the dependent variable was statistically significant only in Model 1 (*b* = 0.249, *p* < 0.05), however, statistical significance is lost with the addition of the core independent variable, and similar results are seen in the role of age and party membership for the dependent variable.

The above research hypotheses were tested through Models 2, 3, 5, and 6. Regarding the core circle, in the rural sample, the effect of the number of daughters on the dependent variable passed the *p* < 0.05 significance test in both Model 2 and 3 and was in the positive direction, indicating that an increase in the number of daughters significantly increased the mean SWB score of rural WOA. The direction of the effect of the number of sons on the dependent variable, although in the same direction as predicted by the hypothesis (*b* = 0.014), failed to meet the statistical significance requirement of *p* < 0.05; In the urban sample, the effect of the number of daughters on the dependent variable was significant and in a positive direction at the *p* < 0.01 level (*b* = 0.114), and this effect remained significant (*p* < 0.05) in Model 6 after the inclusion of control variables, with each increase in the number of daughters resulting in a significant increase of 0.083 in the mean score of the SWB for the urban WOA; The effect of the number of sons on the dependent variable is significant and in a positive direction in Model 5 (*b* = 0.096, *p* < 0.05), but its effect loses statistical significance after the inclusion of the control variable, so we conclude that there is insufficient evidence to support the effect of the number of sons on urban WOA. For the middle circle, in the rural sample, the direction of the effect of frequency of interaction with relatives on the dependent variable was positive in both Model 2 and 3, and its effect was significant at the *p* < 0.001 level, based on Model 3, which showed that for each unit increase in the frequency of interaction with relatives, the mean SWB score of rural WOA significantly increased by 0.17; The increase in the frequency of interaction with friends failed the significance test at *p* < 0.05 in both models, so there is insufficient evidence to support the effect of the frequency of interaction with friends on the SWB of rural WOA. In the urban sample, although the effect of frequency of interaction with relatives on the dependent variable passed the significance test at *p* < 0.01 and was in the positive direction (*b* = 0.132), this effect lost statistical significance in Model 6 with the inclusion of control variables, and the results failed to support the ability of frequency of interaction with relatives to influence SWB among urban WOA; The effect of frequency of interaction with friends on the dependent variable was significant (*p* < 0.05) and in the positive direction in both models, suggesting that an increase in the frequency of interaction with friends has a significant and positive contribution to the SWB of urban WOA. For the outer circles, in the rural sample, the effect of frequency of interaction with neighbors on the dependent variable was significant and positive in both Model 2 and 3, so that an increase in the frequency of interaction with neighbors could significantly increase the mean SWB score of rural WOA; The effect of frequency of religious activity participation on the dependent variable did not pass the *p* < 0.05 significance test in either model, so there was insufficient evidence to support its effect on SWB among rural WOA. In the urban sample, based on the results of Models 5 and 6, which showed that neither the frequency of interaction with neighbors nor the frequency of participation in religious activities had a statistically significant effect on the dependent variable, the present study concludes that there is insufficient evidence to support a significant effect of the above two variables on the SWB of urban WOA.

## 5 Discussion

### 5.1 Urban–rural differences in SWB

An increase in the number of daughters, but not the number of sons, can significantly increase the SWB of urban and rural WOA. This paper argues that the above findings may be related to the filial support of daughters for their older adult parents. Zhan’s study found that daughters have become the main supporters of older adult parents in urban societies (61), in addition, married daughters in urban societies provide higher financial support to older adult parents than married sons (62). Rural-based studies have found that daughters provide more SS to older adult parents, and daughters visit their parents at home more frequently than sons, so most older adult parents tend to believe that daughters are more filial than sons (63). Not only that, related studies have found that daughters are a more important source of emotional support for female WOA (45). Although filial piety has always been considered a higher requirement for Chinese men, daughters tend to provide more emotional support to their aging parents. This is because, on the one hand, more of the day-to-day care for parents comes from daughters, and on the other hand, daughters are better at expressing their closeness to their parents (61, 63). Considering the high correlation between emotional support and SWB (19), daughters in the core circle are more likely to be the guardians of SWB among urban and rural WOA.

The increased frequency of interactions with relatives and neighbors significantly increased the SWB of rural WOA, while the increased frequency of interactions with friends significantly increased the SWB of urban WOA (51). These results may be due to the urban–rural differences in perceptions and patterns of interaction, as rural older adults are relatively less mobile and therefore have closer relationships with relatives and neighbors, whose interactions provide more SS for older adults, and who serve as guardians of SWB for WOA in rural areas. Urban older adults are more likely to form interest groups with friends who have similar personalities and hobbies, and the intimacy, understanding, and respect that come from interacting with friends, make friends the guardians of SWB for urban WOA. In addition, this paper fails to support the existence of a correlation between the frequency of religious activity participation and SWB in WOA, which may be due to two reasons: on the one hand, related studies have found that the correlation between religious activity participation and SWB is affected by some mediating variables (e.g., loneliness, etc.) (64); On the other hand, Chinese beliefs are closely related to ancestor worship, which was not presented in the questionnaire, so the question on religious participation did not include activities such as rituals, thus limiting the explanatory power of religious activity participation for SWB in WOA.

### 5.2 SS and SWB

We summarize all the results in Table 4, using “P” to indicate that a particular type of SS has a significant positive contribution to the dependent variable.

**Table 4:** SS and SWB among WOA.

SS		Core circle		Middle circle		Outer circle	
		Daughters	Sons	Relatives	Friends	Neighbors	Religious
Sample	Rural	P		P		P	
	Urban	P			P		

In the core circle, an increase in the number of daughters significantly raised the SWB scores of urban and rural WOA, and the number of sons had no significant effect on the SWB of urban and rural WOA, so the data supported Hypothesis 1 rather than Hypothesis 2. In the middle circle, an increase in the frequency of interactions with relatives significantly increased the SWB scores of rural WOA, and an increase in the frequency of interactions with friends significantly increased the SWB scores of urban WOA, thus Hypothesis 3 was supported in the rural sample and Hypothesis 4 was supported in the urban sample. In the outer circle, an increase in the frequency of interaction with neighbors significantly enhances SWB scores of rural WOA and the frequency of participation in religious activities has no significant effect on the SWB of urban and rural WOA, Hypothesis 5 is supported in the rural sample and the data fails to support Hypothesis 6.

### 5.3 Limitations and implications

This study has several limitations. First, although we classified the SS of WOA into three types of circles based on the convey model, some potentially important caregivers could not be operationalized in this study, such as siblings, colleagues, etc., considering the limited content of the questionnaire. Second, Since the questionnaire did not include information on intergenerational communication, we used the number of children to reflect the structural characteristics of the core circles, and this operationalization has some limitations. We believe that children must develop a good interactive relationship with their parents. This is because, through effective communication and interaction, understanding and trust between children and parents will be further strengthened, thus providing a safe, supportive, and harmonious environment for WOA to live in their later years. Third, established studies have found that older adults’ subjective assessment of relationship quality is a significant predictor of their SWB (65); unfortunately, we were not able to include this indicator in our analytic model due to the lack of relevant information. Therefore, future research should further examine the effects of the structure and quality of SS on SWB among WOA, based on improving the aforementioned shortcomings.

Despite these limitations, this study is beneficial to the literature on the relationship between SS and SWB. This paper advances related research in two main ways. First, we creatively apply the convey model to the empirical study of SS and SWB, which provides a theoretical model for future related research. Second, this paper presents for the first time the role of different circles of SS on SWB among urban and rural WOA, which not only enriches the relevant empirical research results but also sheds light on the reconstruction of SS for WOA at the practical level.

## 6 Conclusion

Older adults with low SS experience more negative events and daily distress than older adults with more SS, and it has been found that protective resources provided by guardians not only serve as a buffer when older adults are in distress but also reduce the likelihood of negative events occurring, which can enhance older adults’ SWB (17, 66). For WOA, exploring caregivers other than their spouses becomes crucial to safeguarding their SWB, and this paper finds that daughters, neighbors, and relatives constitute the guardians who safeguard the SWB of rural WOA, while daughters and friends constitute the guardians who safeguard the SWB of urban WOA. The above results partially corroborate the findings of established studies on the topic of SS and SWB among older adults, such as the positive utility of child and neighbor support (67); The other part of the results is inconsistent with previous studies and shows the specificity of the WOA group: unlike the results of the significant negative utility of relative interactions on older adults’ SWB, this paper finds a positive facilitating effect for WOA; In addition, this paper supports the positive facilitating effect of interaction with friends on SWB in urban WOA, a finding that, while inconsistent with findings in studies related to SWB in older adults, is consistent with findings in studies related to SWB in urban residents (68), which underscores the role of social situation.

## Data availability statement

The original contributions presented in the study are included in the article/supplementary material, further inquiries can be directed to the corresponding author.

## Ethics statement

Ethical approval was not required for the study involving humans in accordance with the local legislation and institutional requirements. Written informed consent to participate in this study was not required from the participants or the participants' legal guardians/next of kin in accordance with the national legislation and the institutional requirements.

## Author contributions

JZ: Writing – original draft, Writing – review & editing. FC: Conceptualization, Data curation, Resources, Writing – review & editing. CY: Resources, Writing – review & editing.
